# Plant Responses to Vegetation Proximity: A Whole Life Avoiding Shade

**DOI:** 10.3389/fpls.2016.00236

**Published:** 2016-02-29

**Authors:** Irma Roig-Villanova, Jaime F. Martínez-García

**Affiliations:** ^1^Centre for Research in Agricultural Genomics (CRAG), Consejo Superior de Investigaciones Científicas – Institut Recerca i Tecnologia Agroalimentaries – Universitat Autònoma de Barcelona – Universitat de BarcelonaBarcelona, Spain; ^2^Institució Catalana de Recerca i Estudis Avançats (ICREA)Barcelona, Spain

**Keywords:** shade avoidance syndrome, *Arabidopsis thaliana*, hypocotyl, petiole, elongation, adult tissues, reproductive tissues

## Abstract

In high density of vegetation, plants detect neighbors by perceiving changes in light quality through phytochrome photoreceptors. Close vegetation proximity might result in competition for resources, such as light. To face this challenge, plants have evolved two alternative strategies: to either tolerate or avoid shade. Shade-avoiding species generally adapt their development by inducing hypocotyl, stem, and petiole elongation, apical dominance and flowering, and decreasing leaf expansion and yield, a set of responses collectively known as the shade avoidance syndrome (SAS). The SAS responses have been mostly studied at the seedling stage, centered on the increase of hypocotyl elongation. After compiling the main findings about SAS responses in seedlings, this review is focused on the response to shade at adult stages of development, such as petioles of adult leaves, and the little information available on the SAS responses in reproductive tissues. We discuss these responses based on the knowledge about the molecular mechanisms and components with a role in regulating the SAS response of the hypocotyls of *Arabidopsis thaliana*. The transcriptional networks involved in this process, as well as the communication among the tissues that perceive the shade and the ones that respond to this stimulus will also be briefly commented.

## Introduction

Over the past few decades, improvements in crop yields have come largely through increasing planting densities. Under the current scenario of rapid population growth and limited amount of arable land on Earth (http://www.fao.org/news/story/en/item/35571/icode/), efficient agricultural practices might require even higher planting densities together with changes in plant architecture as a way to maximize crop yield. The optimum plant density of each plant species is determined by its peculiar set of requirements and the environmental factors that directly influence plant growth and, consequently, crop yield. However, in general, as planting densities increase so does the shade signals provided by plants and, hence, the activation of plant responses to vegetation proximity. For many plant species, perception of plant proximity results in a set of responses known as the SAS that strongly affects plant development and architecture. When activated, SAS results in a reduced seed set and altered fruit development. Crops are typically grown at high densities, and SAS responses, if activated, are generally detrimental to yield because they induce reallocation of resources into elongation growth at the expense of harvestable organs (usually leaves, fruits, and seeds). Therefore, one key research challenge that will certainly have a major impact in agriculture is to identify the mechanisms that underlie plant development in response to vegetation proximity.

The aim of this review is to describe what is known about the response to shade in adult tissues, such as petioles of adult leaves, and the little information available on the SAS responses in reproductive tissues. We focus on studies performed in *Arabidopsis thaliana* in response to signaling by red (R, about 600–700 nm) and FR (about 700–800 nm), as the influence of PAR (between 400 and 700 nm) and other environmental factors, such as green and blue light, ethylene production or mechanical stimuli have been reviewed recently (Pierik and de Wit, 2014). We discuss the responses occurring in adult tissues based on the knowledge available on the molecular mechanisms and components with a role in regulating the SAS response of the hypocotyls and their participation in the transcriptional networks that mediate this process.

## Vegetation Proximity Signals And Plant Perception

In both natural and agricultural plant communities, light might become a limiting resource under high plant density. In such a situation, plants have evolved to either tolerate or avoid shade. Shade tolerance is achieved by different sets of responses in different species, such as alterations in leaf physiology, biochemistry, anatomy and morphology, and/or plant architecture. In general, under low light intensities, shade tolerant species tend to adapt to a highly conservative utilization of resources, commonly accompanied by low growth rates, thinner leaves, reduced apical dominance (increased branching) and low elongation response (Martinez-Garcia et al., 2010; Casal, 2012; Gommers et al., 2013). By contrast, shade-avoiding (or sun-loving) species generally tend to adapt their development to favor elongation at the expense of leaf development, and to increase apical dominance (reduced branching), allowing young and growing tissues to escape from shade. These SAS responses can be viewed as the optimum strategies to adapt to eventual shading in natural environments. By contrast, activation of SAS is generally detrimental for crop yield in agronomical terms.

### How Vegetation Announces Itself: the R:FR Signal

In open conditions, i.e., when a plant grows under low vegetation density, the light coming from the sun (or sunlight) is relatively constant in quality, and the R:FR is about 1.2–1.5. In environments of high vegetation density, two related but different situations that lower the R:FR can occur: *plant proximity* (without direct shading by neighboring plants) and *direct plant canopy shade*. Because vegetation is considered to preferentially reflect FR compared to other wavelengths, plant proximity generates an intermediate or moderate reduction in the R:FR. By contrast, under a plant canopy, photosynthetic pigments from green tissues specifically absorb light from the PAR region (between 400 and 700 nm) whereas FR, which is poorly absorbed by plant tissues, is reflected from or transmitted through vegetation. Consequently, under direct plant canopy shade both the amount of PAR and R:FR is strongly reduced (low and very low R:FR), the latter effect mostly due to the selective depletion of the R of the sunlight filtered by the leaves (Smith, 1982; Martinez-Garcia et al., 2010; Casal, 2013).

### How Plants Perceive Nearby Vegetation: The Role of Phytochromes

The R:FR changes indicative of plant proximity or shade are detected by the phytochrome photoreceptors, which in *A. thaliana* are encoded by a small gene family of five members (*PHYA* to *PHYE*). Whereas *PHYA* encodes the only photolabile phytochrome, phyA, the other *PHY* genes encode photostable phytochromes, phyB–phyE. Phytochromes exist in two photoconvertible forms, an inactive R-absorbing Pr form and an active FR-absorbing Pfr form. Consequently, in the light, the steady-state ratio of Pr and Pfr forms (that can also be described as the relative levels of active Pfr form, Pfr/Ptot) depends on the R:FR. Indeed, there is a positive correlation between the R:FR and the Pfr/Ptot levels. Under high R:FR (i.e., low vegetation density) the photoequilibrium is displaced toward the active Pfr form, the Pfr/Ptot is high (0.7 or higher), and the SAS is suppressed. On the contrary, under low R:FR the photoequilibrium is displaced toward the inactive Pr form, the Pfr/Ptot is low (0.6 or lower), and the SAS is induced (Smith, 1982, 2000; Casal, 2012, 2013; Possart et al., 2014).

**FIGURE 1:**
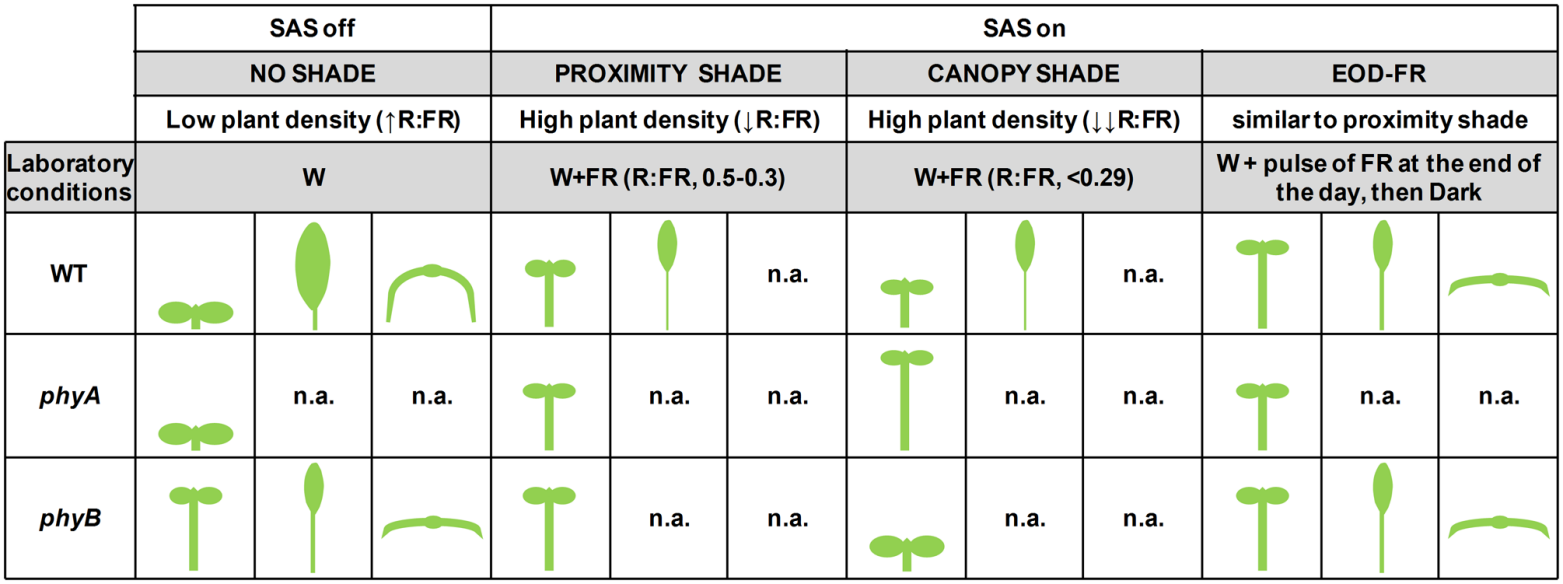
**Summary of phenotypes displayed by wild-type, *phyA* and *phyB* mutant plants grown under different light conditions.** Seedling (hypocotyl elongation) and leaf phenotypes (petiole elongation, blade expansion and leaf curling) are shown for each of the genotypes and light treatments indicated. Cartoons summarize the results presented by different authors mentioned in this review. n.a., data not available; WT, wild type.

Genetic analyses in *Arabidopsis* have shown that mutants deficient in phyB display long hypocotyls and early flowering when growing under high R:FR (in open conditions or under low density of vegetation). These are phenotypes very similar to those of wild-type plants growing under low R:FR (**Figure 1**). Therefore, the constitutive SAS phenotype of *phyB* mutant plants was taken as indicative that phyB is repressing SAS responses under high R:FR conditions, i.e., that responses to low R:FR are primarily mediated by phyB. Mutant analyses also showed that phyD and phyE act redundantly with phyB in controlling adult SAS responses, such as petiole elongation and internode elongation between rosette leaves (reviewed in Martinez-Garcia et al., 2010; Casal, 2012).

Genetic analyses have also indicated that *phyA* mutant seedlings display enhanced (rather than reduced) responses to low R:FR, which indicates that phyA and phyB have antagonistic actions in shade. This is particularly obvious when growing under deep plant shade. Whereas vegetation proximity (that results in moderate R:FR) promotes a similar hypocotyl elongation in wild-type and *phyA* seedlings, under deep and direct plant canopy shade (that results in low and very low R:FR) hypocotyls of wild-type seedlings tend to elongate significantly less than those of *phyA* mutant seedlings, which display an exaggerated elongation (**Figure 1**; Johnson et al., 1994; Yanovsky et al., 1995; Martinez-Garcia et al., 2014; Possart et al., 2014). Therefore, phyB is deactivated by shade of intermediate, low and very low R:FR, resulting in a promotion of hypocotyl elongation. By contrast, phyA is strongly activated mostly by shade of low and very low R:FR, conditions that prevent the excessive elongation of the seedling caused by the deactivation of phyB, hence ensuring an optimum hypocotyl elongation under deep shade. Therefore, phyA and phyB actions help to distinguish between plant proximity and direct plant canopy shade (Martinez-Garcia et al., 2014).

### Mimicking Plant Proximity and Shade in the Laboratory with Light Treatments: Low R:FR and End-of-Day-FR (EOD-FR) Treatments

Laboratory conditions that mimic plant proximity, before actual canopy shading occurs, have been termed *simulated shade*, and those that mimic natural direct plant shade, when canopy closure occurs, *canopy shade* (Roig-Villanova et al., 2007; Possart et al., 2014). Several authors, however, employ the term *simulated shade* as synonymous of intermediate, low or very low R:FR treatments, i.e., independently on the amount of FR employed. To avoid ambiguities and to compare the different conditions, in this paper we will employ the terms (1) **simulated shade,** to refer to any conditions that lower the R:FR and have not been defined by the authors as mimicking vegetation proximity or shade; (2) **proximity shade,** to those conditions that simulate plant or vegetation proximity; and (3) **canopy shade,** to those conditions that mimic natural direct plant shade. These treatments are obtained in the laboratory by applying increasing intensities of FR to a fixed intensity of white light (W), usually provided by fluorescent tubes: for a given amount of PAR, intermediate R:FR (about 0.50–0.30) results in proximity shade treatments, and low and very low R:FR (<0.29) results in canopy shade treatments (Martinez-Garcia et al., 2014). These R:FR values, however, might differ between laboratories depending on the window (in nm) of the R and FR measured. A better way to distinguish or compare these shade conditions, as already discussed before, is genetically by analyzing the differential hypocotyl elongation of the *phyA* and *phyB* mutant seedlings (**Figure 1**).

One might think that the very low R:FR of the canopy shade treatments rarely exists in nature: because the amount of FR employed in the laboratory to supplement the W is very high, it seems difficult to comprehend how nature can produce such as high proportion of FR simply by filtering and transmitting differentially the R and FR components of the sunlight. In that respect it is important to keep in mind that in chlorophyll-rich organs, such as leaves, light absorption from the ultraviolet to the R part of the visible region by chlorophyll leads to the emission of FR by fluorescence (Thornber, 1975), a property that might contribute to create low and very low R:FR signals in natural dense canopies. Indeed, there are reports for canopies in which R:FR of less than 0.10 have been measured (reviewed in Smith, 1982).

In addition to supplementing W with FR, an alternative way to induce SAS responses is to treat plants with a pulse of FR for a few minutes at the end of the light period and immediately before starting the dark phase of the photoperiod. These are called end-of-day-FR (EOD-FR) treatments (Smith, 1982). Like low R:FR, the EOD-FR treatments reduce the Pfr active form of the phytochromes during the subsequent dark period. These treatments are less representative of the natural neighbor signals, although they are considered to induce those shade responses dependent on the photostable phytochromes (Johnson et al., 1994), i.e., those promoted by vegetation proximity.

## Shade Avoidance Responses In Seedlings

### The Response of Seedlings to Plant Proximity and Shade: Looking for Simplicity Inside Complexity

Plants at seedling stage have been shown to respond to shade at the level of hypocotyls/epicotyls, cotyledons and primary leaves. While the cotyledon (petiole and area) response to simulated shade has been poorly studied, hypocotyl elongation has been most commonly and deeply studied. Analyses of the hypocotyl elongation responses to simulated shade in *Arabidopsis* led to conclude that the SAS induction is regulated at least in part by the interaction of active phytochromes with the PIFs (Leivar and Quail, 2011; Jeong and Choi, 2013), which results in rapid changes in the expression of dozens of *PHYTOCHROME RAPIDLY REGULATED* (*PAR*) genes, postulated to be instrumental for the implementation of the SAS responses. Because many of these *PAR* genes encode transcriptional regulators, it is considered that SAS responses are consequence of the phytochrome regulation of a transcriptional network, composed by several types of transcriptional regulators organized in partially redundant functional modules. Genetic analyses performed measuring the hypocotyl elongation demonstrated roles in the SAS regulation for several *PAR* genes. This is the case of members of the HD-Zip II, (e.g., ATHB2, ATHB4, HAT1, HAT2, and HAT3), bHLH (e.g., BEE1, BIM1, HFR1, PAR1, and PIL1) and B-BOX CONTAINING (BBX) families of proteins. Analyses of mutants with altered levels of these factors led to propose negative (HFR1, PAR1, PIL1, BBX21, and BBX22), positive (BEEs, BIMs, BBX24, and BBX25) and complex (HD-Zip II) roles in the SAS regulation (Salter et al., 2003; Sessa et al., 2005; Roig-Villanova et al., 2006, 2007; Hornitschek et al., 2009; Sorin et al., 2009; Crocco et al., 2010; Cifuentes-Esquivel et al., 2013; Gangappa et al., 2013). Further analyses have also involved non-PAR factors in the regulation of SAS hypocotyl response such as HD-Zip class III and growth repressing DELLA proteins (Djakovic-Petrovic et al., 2007; Brandt et al., 2012). PIF1, PIF3, PIF4, PIF5 (called the PIFQ), and PIF7, proteins of the bHLH family known to promote growth, were also identified as positive players of the hypocotyl SAS response (Leivar et al., 2012; Li et al., 2012). In contrast with *PAR* genes, the expression of these *PIF* genes is unaffected by simulated shade, but the phosphorylation of PIF proteins is promoted by the active form of phytochromes, which reduces PIFQ stability and PIF7 DNA-binding activity. Consequently, the reduction in the levels of active phytochromes under low R:FR increases the stability of PIFQ and the DNA-binding activity of PIF7 to its target genes, which results in the up-regulation of *PAR* expression (Lorrain et al., 2008; Li et al., 2012). It seems, therefore, that simulated shade perception rapidly changes the balance of positive and negative factors that eventually cause hypocotyls to elongate.

**FIGURE 2:**
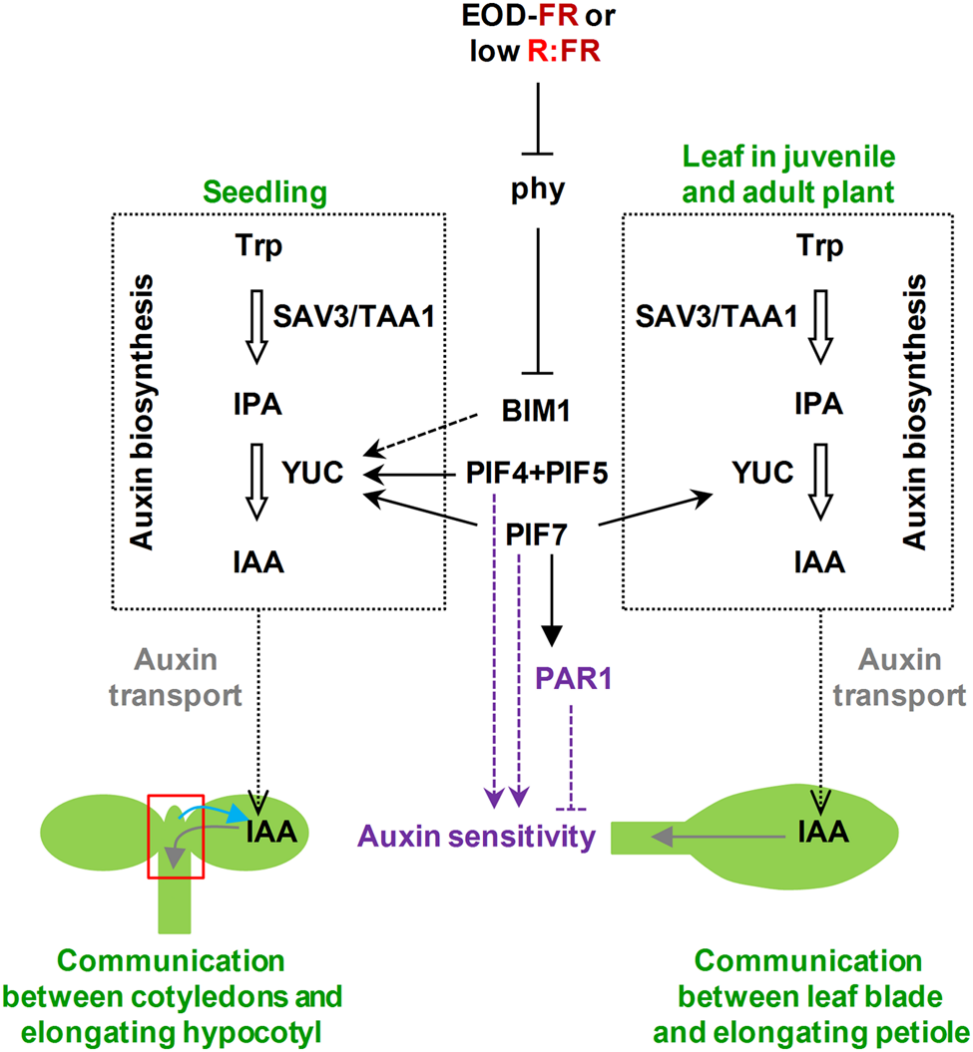
**Scheme to explain how phytochrome action may regulate auxins (biosynthesis, transport, and sensitivity) in the response of seedlings and leaves of adult plants to plant proximity.** Shade perception integrates with auxins at multiple levels, affecting both auxin biosynthesis and sensitivity. It is unknown whether shade perception also affects auxin transport. The two steps in auxin biosynthesis (enclosed in black dotted squares and represented by thick empty arrows) are indicated together with the involved enzymes (SAV3/TAA1 and YUC). Auxin transport is indicated in gray and auxin sensitivity in purple. Black and purple lines indicate that the role of known regulators may be either direct (continuous) or indirect (discontinuous), and positive (arrow heads) or negative (flat ends). Gray arrows refer to the direction of IAA transport in seedlings or leaves, whereas the blue arrow indicates the signal from the shoot apex (area delimited by the red square) toward the cotyledons suggested by Nito et al. (2015). Trp, Trytophan; IPA, indole-2-piruvic acid; IAA, indole-3-acetic acid.

Simulated shade treatments were shown to rapidly and transiently increase endogenous levels of auxins (Tao et al., 2008; Hornitschek et al., 2012; Li et al., 2012; Bou-Torrent et al., 2014). *SAV3* encodes TAA1, an enzyme required for the shade-induced biosynthesis of IAA, a bioactive auxin (Tao et al., 2008). *YUC* genes encode flavin monooxygenase-like proteins that catalyze a rate-limiting step in IAA biosynthesis. Indeed, TAA and YUC families function in the same auxin biosynthetic pathway, known as the indole-2-pyruvic acid (IPA) pathway (**Figure 2**), which produces IAA that is essential for plant development (Mashiguchi et al., 2011; Zhao, 2014). PIF4, PIF5, and PIF7 were shown to directly regulate the shade-induced expression of some *YUC* genes; in addition, the shade-mediated increase of auxin was much reduced both in *pif4pif5* and *pif7* mutant seedlings. These results led to propose that PIF4, PIF5, and PIF7 might directly control auxin biosynthesis. PIFs also directly regulate auxin responsive genes, such as *INDOLE-3-ACETIC ACID INDUCIBLE 29* (*IAA29*), indicating as well a possible involvement of PIFs in modulating auxin sensitivity (Hornitschek et al., 2012; Li et al., 2012). Genetic analyses indicate that BIM transcription factors might also play a direct or indirect role in auxin synthesis via the control of *YUC* expression (Cifuentes-Esquivel et al., 2013). In addition, several PAR factors, such as ATBH4, PAR1, BIM, and BEE proteins, provide entry points by which shade- and auxin-regulated networks are integrated (Sorin et al., 2009; Bou-Torrent et al., 2014). In summary, there is abundant evidence for the involvement of several factors in controlling auxin levels and sensitivity in the hypocotyl elongation response. However, how auxin and other hormones are integrated in controlling shade-induced hypocotyl elongation is currently unclear.

### The Physical Separation Between the Sites of Shade-Perception and Response: Inter-Organ Communication

There is an additional spatial level of regulation of seedling responses to SAS. The physical separation between the sites of shade perception and response is highlighted by a miscellany of experimental approaches in different species that employed localized shade irradiation (EOD-FR or low R:FR) of cotyledons (or primary leaves, as we will see later) combined with molecular or physiological analyses in the responding stems (hypocotyls or epicotyls; reviewed by Bou-Torrent et al., 2008).

Thus, detailed analyses in *Brassica rapa* seedlings suggested that shade perception in the cotyledons, the site of photoperception of this signal in the seedlings, triggers local synthesis of IAA that is then transported to the hypocotyl. In there, auxin induces the up-regulation of auxin-related genes associated with the SAS hypocotyl response. Together, the results show that cotyledon-generated auxin regulates hypocotyl elongation (Procko et al., 2014). Organ-specific transcriptomic analyses using micro-samples prepared from the topmost part of the hypocotyls (referred to as shoot apex samples) and cotyledons from *Arabidopsis* young seedlings differentially irradiated with or without EOD-FR indicated that the response to shade in terms of changes in gene expression in the shoot apex is more prominent than in the cotyledons, likely reflecting the higher diversity of cell types in the shoot apex samples. The finding of specific and shared EOD-FR-induced genes in both types of samples suggests the existence of tissue specific and common regulatory components involved in the implementation of the SAS responses in the different tissues. In addition, localized EOD-FR spotlight irradiation also indicated that EOD-FR induced gene expression depends on both organ-autonomous (i.e., *HFR1* and *ATHB2*) and interorgan mechanisms, which involve the role of auxins and PIFs. Indeed, cotyledons are the major site of shade perception that controls several apex-responsive genes, including those that are auxin-responsive and/or PIF7-dependent. This is consistent with the known role of PIF7 predominantly regulating the seedlings SAS response by controlling auxin biosynthesis in cotyledons (**Figure 2**). However, the shade signal perceived in the shoot apex also controls gene expression in the cotyledons (i.e., *YUC2*), providing evidence for an unexpected bidirectional communication between these seedlings parts (Nito et al., 2015). Although the physiological relevance of the two-way communication is unclear, it seems likely that this is part of a feedback mechanism to coordinate the response of the different organs of the seedlings.

## Shade Avoidance Responses In Adult Plants

In seedlings, low R:FR promotes elongation of the petiole of cotyledons in *Arabidopsis* and inhibits cotyledon expansion in *B. rapa* (Roig-Villanova et al., 2007; Procko et al., 2014). In plants that present a non-rosette habit of growth, such as cowpea, tobacco, or mustard, internode elongation is also strongly promoted in response to a variety of shade-mimicking treatments (Casal and Smith, 1988; Martinez-Garcia et al., 2000; Pierik et al., 2004). In an even more complex way than in seedlings, *Arabidopsis* shows a broad range of responses to shade in the adult tissues such as promotion of petiole elongation in the rosette leaves (usually together with an inhibition of leaf blade expansion), promotion of flowering and inflorescence stem length together with a suppression of axillary bud outgrowth (Finlayson et al., 2010; Gonzalez-Grandio et al., 2013). In this section, only leaf responses will be covered, including the effects on leaf polarity. We will also discuss the role of shade on the reproductive development.

### Petiole Elongation, Leaf Expansion, and Leaf Curling

**FIGURE 3:**
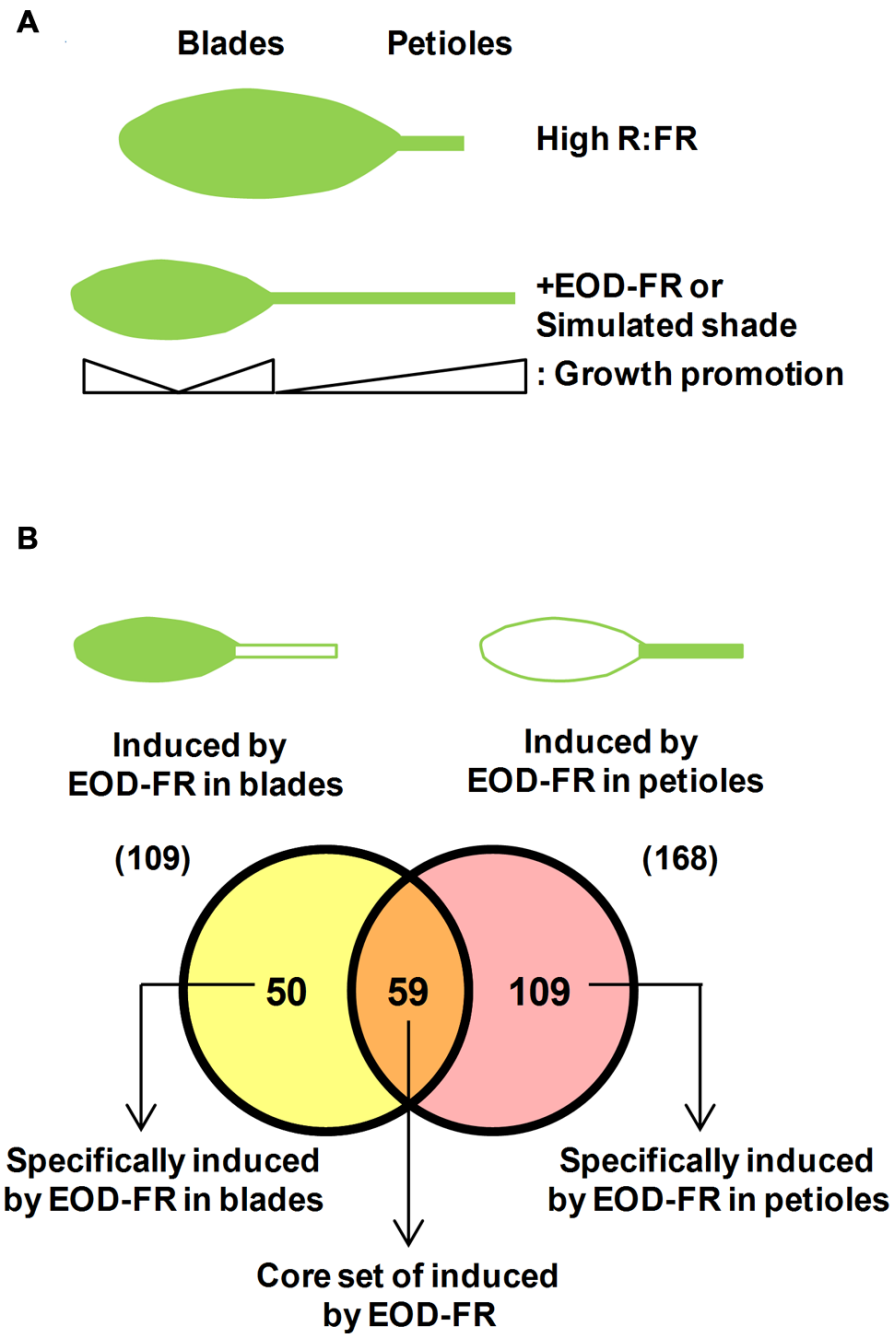
**Adult leaves present a dual response to plant proximity and shade. (A)** Cartoon representing the two parts that form an *Arabidopsis* adult leaf and their differential growth response to EOD-FR or simulated shade treatments: growth promotion of the petiole length and growth inhibition of the blades. **(B)** A Venn diagram illustrating differences in the expression of genes induced by EOD-FR in blades and in petioles allows identifying a core set of genes that are induced in both tissues. Data are extracted from available microarray data (Sasidharan and Pierik, 2010).

The *Arabidopsis* leaf consists of a blade (or lamina) and a petiole (**Figure 3A**). The petiole places the leaf blade in the position that is best suited for light reception. Hence, the regulation of petiole elongation seems important to maximize photosynthesis. One of the most studied SAS response of the rosette leaves of *Arabidopsis* is petiole elongation, which is promoted by light treatments that simulate vegetation proximity or canopy shade, such as low R:FR and EOD-FR (Kozuka et al., 2005; Sasidharan et al., 2010; **Figures 1** and **3A**). Genetic analyses indicate that leaf petiole elongation is regulated by photostable phytochromes: the *phyB* mutant has longer petioles than the wild type (**Figure 1**), and the *phyAphyBphyD* and *phyAphyBphyE* mutants have longer petioles than the *phyAphyB* double mutant (Reed et al., 1993; Devlin et al., 1998). Therefore, the promotion of petiole growth by shade signals is mediated mainly by phyB with the contribution of phyD and phyE (Casal, 2012).

Growth and elongation of plant organs require the normally rigid cell wall to weaken and thus allow continued expansion. XTHs are well-characterized cell wall modifying proteins that have been involved in cellular expansion (Rose et al., 2002). Indeed, petiole elongation is associated with an increase in XTH activity, which acts enzymatically (hydrolysis and/or transglucosylation) on the cellulose and hemicelluloses (xyloglucan in most eudicots) network and results in the necessary cell wall loosening. In *Arabidopsis*, XTHs are encoded by a large multigene family, and the expression of a few of them (*XTH9*, *XTH15/XTR7*, *XTH16*, *XTH17*, and *XTH19*) is up-regulated by shade. However, only the knockout mutants for *XTH15* and *XTH17* had reduced or absent petiole SAS responses. Together, these results confirm the functional role of some XTHs in the cell wall restructuration required for the cellular expansion that fuels rapid petiole elongation during the SAS (Sasidharan et al., 2010).

In addition to the promotion of petiole elongation, the growth and expansion of leaf blades is suppressed under both simulated shade conditions and EOD-FR treatments (**Figures 1** and **3A**; Kozuka et al., 2010; de Wit et al., 2015). The two antagonistic effects of shade on leaf growth are very likely concomitant and connected (**Figure 3A**). This suggests that when leaves are exposed to proximity or canopy shade, growth is invested in petiole elongation at the expense of blade expansion. The EOD-FR treatment promotes more strongly the elongation of the basal part of the petiole, although both the basal and apical part of this organ elongate. Similarly, the EOD-FR inhibited mostly the expansion of the middle portion of the leaf blades (**Figure 3A**). At the leaf stage analyzed, both effects happened by differential control of cell elongation, with a negligible contribution of cell division (Kozuka et al., 2005). Other authors showed that the reduced blade area of the first leaf fully grown under canopy shade and reduced light intensities is caused by decreased cell proliferation rather than cell size (Carabelli et al., 2007). These opposed observations might account for the different shade treatments and age of the leaves on which shade was applied by different laboratories, and suggest that exposure to proximity or canopy shade might affect primarily cell division at early stages of leaf development and mainly cell expansion in already formed leaves. Mutant *phyB* plants display elongated petioles and smaller leaf blades compared to wild-type plants, a phenotype that indicates a role for phyB in the regulation of these traits in opposite manners (**Figure 1**; Robson et al., 1993; Kozuka et al., 2005).

It seems, therefore, that the shade similarly affects the pattern of growth of photosynthetic organs (i.e., it inhibits leaf blade and cotyledon expansion) and of supportive organs (i.e., it promotes leaf petiole and hypocotyl elongation). It is unknown whether the regulators of shade-induced hypocotyl elongation also control the shade-induced petiole elongation. Available transcriptomic data suggest the existence of some common regulators (Kozuka et al., 2010). Microarray analyses revealed that many auxin- and brassinosteroid (BR)-responsive genes showed altered expression in response to EOD-FR in both the leaf blade and the petiole. Indeed, 52 and 81 auxin-responsive genes, including the *IAA*, *GH3* and *SMALL AUXIN UPREGULATED RNA* (*SAUR*) families, were up-regulated by EOD-FR in the leaf blade (from a total of 109 genes) and petiole (from a total of 168 genes), respectively. Genes that are dually controlled by auxin and BR were overrepresented in the EOD-FR-induced genes also in leaf blades and petioles (Kozuka et al., 2010). Similarly, rapid changes in the expression of several auxin- and BR-responsive genes were observed in simulated shade exposed seedlings (Bou-Torrent et al., 2014). Amongst the genes induced by the EOD-FR treatments in petioles and/or blades, several were found to encode transcriptional regulators known to be instrumental for the SAS seedling response, such as *ATHB2*, *HAT2*, *HAT3*, *HFR1*, *PAR1*, *BEE1*, and *BIM1* (**Figure 3**; Supplementary Table S1). Some of these genes are directly regulated by PIFs (e.g., *ATHB2*, *HFR1*, and *PAR1*), suggesting that these factors also have a regulatory role in the shade-induced leaf growth and expansion. Although there is not yet experimental evidence confirming this possibility, single or multiple *PIF*-deficient mutants, such as *pif4* and the double *pif4pif5* show small and compact rosettes; in agreement, *PIF4* and *PIF5* overexpressing plants display expanded rosettes with strongly elongated petiole leaves under a range of light conditions or temperatures (Pierik et al., 2004; Lorrain et al., 2008; Sasidharan et al., 2010). Recent advances indicated that PIF7 has a predominant role in regulating petiole elongation and leaf blade expansion in response to low R:FR, whereas PIF4, PIF5, and HFR1 have a minor or no role in these leaf responses. In addition, auxin levels are induced in the leaf blade shortly after exposure to low R:FR, a burst that is absent in *pif7* mutant (de Wit et al., 2015). These findings supports the existence of both shared (PIF7) and unique (PIF4, PIF5, HFR1) regulatory components and/or mechanisms of the SAS in different stages of development, e.g., hypocotyl and leaf growth.

Perception of shade in rosette plants such as *Arabidopsis*, which have leaves close to the ground, usually results in a repositioning of the leaves, which helps to avoid shading imposed by neighboring plants. The upward bending of the leaves, caused by faster growth on their lower than their upper side, is called hyponasty. The hyponastic response to low R:FR is severely reduced in a range of auxin mutants, such as *sav3*, which encodes an enzyme involved in the rapid increase of endogenous levels of IAA required for the shade-induced hypocotyl elongation (**Figure 2**), and *pin3*, which encodes a protein that controls the direction and rate of cellular auxin eﬄux, indicating that the leaf hyponastic response to simulated shade requires an intact auxin signaling (Casal, 2012).

Proximity or canopy shade seems also to affect an additional aspect of leaf blade development: curling or flattening. The regulation of leaf flatness is considered to contribute to the efficient absorption of light under low light, conditions that accompany the canopy shade. When analyzing wild-type and *phyB* plants grown under continuous W, it was observed that in the wild type, the fourth leaves were gently curled downward whereas in *phyB*, leaves were flat. In transgenic lines overexpressing *PHYB* the leaves were more severely curled than in the wild type, suggesting that phyB inhibits flattening of the leaves (Kozuka et al., 2013). In addition, in the wild type, leaf flattening was promoted by EOD-FR treatments, which rapidly eliminated the active Pfr phytochrome. Whereas *phyB* exhibited very flat leaves without EOD-FR and failed to respond to EOD-FR, the already curled-leaf phenotype in *PHYB* overexpressing plants was substantially suppressed by EOD-FR. Taken together, it was concluded that the active Pfr form of phyB promotes leaf curling, a process that might involve the regulation of auxin responsiveness in this organ (Kozuka et al., 2013).

Leaf curling is most probably caused by an uneven expansion of epidermal cells on the adaxial (upper) and abaxial (lower) leaf sides, although no information is available about the effect of shade on the epidermal cell area on these sides of the leaves. It is intriguing to find that mutants with altered levels of *ATHB4* and/or *HAT3*, two paralog genes encoding transcription factors of the HD-Zip class II and identified as regulators of the SAS hypocotyl elongation response (Sorin et al., 2009), do show a role in controlling the dorso-ventral patterning of leaves. Whereas double *athb4 hat3* mutant plants result in severely abaxialized leaves, overexpression of *HAT3* results in adaxialized leaf development, which is visualized by a strong upward-curling of leaf blades, a phenotype caused by overproliferation of abaxial-derived tissues in leaves (Bou-Torrent et al., 2012). Since long-term exposure to shade results in leaves with flattened blades, these results suggest that *ATHB4* and *HAT3*, shown to be functionally redundant (Sorin et al., 2009), might be part of the mechanisms regulating this SAS response in adult leaves.

In summary, at the rosette stage, shade light signals promote upward positioning of leaves and petiole elongation; usually, they also reduce lamina expansion and enhance flattening of the leaves (**Figure 1**). The combination of these responses places leaf lamina away from the shade of neighbors, likely enhancing the photosynthetic activity of the plant. Current evidence supports that some regulators of these responses could be shared with those in seedlings, despite the structural differences between the involved organs. Very recently it has been also suggested that *Arabidopsis* plants reorient their leaves upon recognition of kin neighbors (Crepy and Casal, 2015), although there is some controversy on whether this is more a case of phenotype matching (Crepy and Casal, 2016; Till-Bottraud and de Villemereuil, 2016). The beneficial effects of these rearrangements on plant fitness by alleviating the strong competition for light open an interesting subject of study.

Spotlight EOD-FR irradiation applied separately to the leaf blade and petiole indicated that the photoperceptive site for the regulation of petiole elongation was the leaf blade but not the petiole itself. A candidate molecule for the mobile signal is, again, auxin. Analyses of expression of *GH3.3* (*At2g23170*) and *IAA6* (*At1g52830*), two auxin-responsive genes in the petiole identified previously as also being induced after EOD-FR treatment of the whole plant, showed that their expression was under the control of phytochrome in the leaf blade; i.e., the spotlight given on the leaf blade effectively induced gene expression not only in the leaf blade but also in the petiole. In contrast, petiole irradiation was not effective at all in inducing the expression of these two genes in the petiole itself. Together, these simple and elegant experiments indicated that phytochrome action in the leaf blade controls gene expression and elongation responses in the petiole (Kozuka et al., 2010). Auxin transport from the blade to the petiole seems to play a role in this elongation response since the auxin transport inhibitor NPA specifically suppresses petiole elongation in response to EOD-FR or to low R:FR (Pierik et al., 2009; Kozuka et al., 2010), even when the NPA was locally applied just in the petiole-blade junction (de Wit et al., 2015). More recently, it was shown that low R:FR treatments rapidly increase IAA levels in the leaf blade, a process dependent on PIF7 (de Wit et al., 2015), a result suggesting that low R:FR-induced auxin production takes place mainly in the leaf lamina and would be then transported to the petiole, a blade-petiole communication that might be analogous to the one of the cotyledon-hypocotyl (Hornitschek et al., 2012; Li et al., 2012; Bou-Torrent et al., 2014). However, the similar kinetics of auxin levels in the blade and petioles of low R:FR-treated plants does not completely fit with this model, suggesting that other aspects, such as changes in auxin sensitivity, may also have a role in this SAS response.

### SAS Responses During Plant Reproduction: A Sophie’s Choice?

*Arabidopsis* plants grown under photoperiodic conditions in which the light phase has a low R:FR display a marked reduction in flowering time. As this response has been recently covered in great detail (Casal, 2012), it will not be discussed in here. Accelerated flowering of *Arabidopsis* plants has been associated with reduced seed set, truncated fruit development, and often a severe reduction of the germinability of the seeds produced (Smith and Whitelam, 1997), although a highly significant genetic variation for the germination response was found (Dechaine et al., 2009). However, there is very little information in *Arabidopsis* or related species about these SAS responses. Recently, it was described that *B. rapa* adult plants grown under simulated shade showed noticeably longer siliques, produced fewer mature seeds per silique, and those seeds were smaller (Procko et al., 2014). *Arabidopsis* plants grown under the same conditions had significantly reduced seed yield per plant (Procko et al., 2014). In W conditions, phyB-deficient mutant plants of *B. rapa* (named as *ein194*) displayed reduced seed number per silique compared with wild-type plants, suggesting that some of these phenotypes are in part determined by phyB signaling. PhyB activity did not appear to play a significant role in the determination of other phenotypes examined, such as silique length and seed weight (Procko et al., 2014). In *Arabidopsis*, the endogenous factors that determine ovule/seed number are starting to be elucidated [reviewed by (Cucinotta et al., 2014)]. However, due to the poor characterization of shade responses in *Arabidopsis* adult reproductive stages, there is virtually no information about its regulation by this environmental signal.

Transcript levels of a few *PAR* genes, such as *PHYB*, *HFR1*, *PIL1*, *ATHB4*, and *HAT3*, were significantly increased under low R:FR conditions in inflorescences, indicating that reproductive tissues perceive and respond (at least molecularly) to plant proximity or shade (Reymond et al., 2012). In seedlings, the expression of *PIL1* and *HFR1* is directly regulated by the action of some PIFs in response to simulated shade (Hornitschek et al., 2012). Hence, it will be interesting to address whether PIFs also have a role in the regulation of the mentioned SAS responses in reproductive tissues.

In general, SAS responses are considered to redirect photoassimilates toward elongation at the expense of storage organs. In reproductive stages, in which no major elongation events are involved, exposure to plant proximity or canopy shade makes plants to face a different dilemma: whether to invest in producing lots of seeds, many of which might not be viable because of the insufficiency of photoassimilates, or in a limited amount of viable seeds. In this Sophie’s choice, plants seem to favor the latter strategy. Finding the regulatory components and mechanisms involved might help to develop novel ways to improve crops for the coming and challenging times.

## Final Remarks

Along this review we have highlighted the idea that in order to adjust plant growth to shade conditions, responses might vary depending on the organ, the stage of development and/or the species considered. These studies have been centered mainly in *Arabidopsis* and related dicots. In monocots, most of the analyzed responses to shade refer to changes in plant architecture, which strongly reduce yield. Indeed, like in *Arabidopsis* and other dicots, a robust SAS responses in grasses could divert resources to stem elongation at the cost of grain production (reviewed by Warnasooriya and Brutnell, 2014).

Despite the efforts made by many laboratories during the last decades to understand the SAS (reviewed in Martinez-Garcia et al., 2010; Casal, 2012; Pierik and de Wit, 2014), there is still little information about these responses in adult plants. There is, for instance, a special lack of knowledge about the photoperceptive site of shade in SAS responses in reproductive tissues. In relation to the shade-promotion of flowering in *Arabidopsis*, it seems likely that the site of shade perception (leaves) is separated from the site of action (shoot apical meristem), as it is known to happen in the photoperiodic-dependent flowering induction. However, there is no information about this aspect in the SAS responses of the reproductive tissues, although, as mentioned, inflorescence tissues perceive and respond molecularly to plant proximity (Reymond et al., 2012). Considering that in *Arabidopsis* when inflorescences are fully growing, rosette leaves are already senescing, it seems likely that – for these responses - the site of perception and action coincide. Thus, future research on the characterization of the SAS responses in adult plant stages, as well as on its spatial and genetic regulation, will greatly contribute to the understanding of this important adaptive trait with critical impact on crop yield.

## Author Contributions

IR-V and JFM-G wrote the manuscript.

## Conflict of Interest Statement

The authors declare that the research was conducted in the absence of any commercial or financial relationships that could be construed as a potential conflict of interest.

## Abbreviations

ATHB: *ARABIDOPSIS THALIANA* HOMEOBOX
BBX: B-BOX CONTAINING proteins
BEE: BR ENHANCED EXPRESSION
bHLH: basic-helix-loop-helix
BIM: BES1-INTERACTING MYC-LIKE
EOD-FR: end of day-far red
FR: far-red light
HAT: HOMEOBOX FROM *ARABIDOPSIS THALIANA*
HD-Zip II: homedomain leucine zipper class II
HFR1: LONG HYPOCOTYL IN FAR-RED1
IAA: indole-3-acetic acid
*IAA* gene: *IAA INDUCIBLE* gene
IPA: indole-2-piruvic acid
NPA: 1-N-naphthylphthalamic acid
PAR: photosynthetic active radiation
*PAR* genes: *PHYTOCHROME RAPIDLY REGULATED* genes
*PHY*: *PHYTOCHROME*
PIF: PHYTOCHROME INTERACTING FACTOR
PIFQ: PIF quartet
PIL1: PIF3-LIKE 1
PIN3: PIN FORMED 3
Pfr: active form of the phytochromes
Pr: inactive form of the phytochromes
R: red light
R:FR: R to FR ratio
SAS: shade avoidance syndrome
*SAUR* genes: *SMALL AUXIN UPREGULATED RNA* genes
SAV3: SHADE AVOIDANCE 3
TAA1: TRYPTOPHAN AMINOTRANSFERASE OF *ARABIDOPSIS* 1
W: white light
XTHs: Xyloglucan endotransglucosylase/hydrolases
*YUC* genes: *YUCCA* genes
