# Supplementary material for: Plant Responses to Vegetation Proximity: A Whole Life Avoiding Shade
Source: Front Plant Sci. 2016 Feb 29;7:236. doi: 10.3389/fpls.2016.00236 (PMC4770057; doi:10.3389/fpls.2016.00236)
Supplement: Supplementary file 1 [file Table_1.PDF]

Table S1. Lists of the EOD-FR-induced genes in leaf blades and petioles. Adapted from Kozuka et al., (2010).

Expressed in PETIOLES ONLY					
**AGI code**	**Description**	**Expressed in petioles?**	**Induction in petioles - EOD-FR/D**	**Expressed in blades?**	**Induction in blades - EOD-FR/D**
At3g60390	HAT3	Yes	2.81	No	-
At3g62090	PIF6/PIL2	Yes	5.40	No	-
At5g39860	PRE1	Yes	9.58	No	-
At3g50060	R2R3-MYB family protein	Yes	2.10	No	-
At3g55730	MYB109	Yes	2.32	No	-
At1g04240	IAA3/SHY2	Yes	3.26	No	-
At1g52830	IAA6	Yes	16.21	No	-
At4g28640	IAA11	Yes	2.70	No	-
At1g78700	BES1/BZR1 homolog protein 4	Yes	2.12	No	-
At1g77640	ERF/AP2 family protein	Yes	4.37	No	-
At5g25190	ERF/AP2 family protein	Yes	5.20	No	-
At1g21910	ERF/AP2 transcription factor family	Yes	4.60	No	-
At3g58120	bZIP transcription factor family protein	Yes	3.91	No	-
At1g69570	Dof-type zinc finger protein	Yes	4.17	No	-
At1g20900	ESC/AHL27	Yes	2.58	No	-
At1g06850	ATBZIP52	Yes	2.12	No	-
At3g24520	HSFC1	Yes	2.36	No	-
At4g01250	WRKY22	Yes	2.26	No	-
At4g34770	SAUR1	Yes	2.31	No	-
At2g21200	SAUR7	Yes	4.30	No	-
At4g38850	SAUR15	Yes	3.35	No	-
At4g13790	SAUR25	Yes	53.66	No	-
At4g12410	SAUR35	Yes	8.45	No	-
At4g34760	SAUR50	Yes	2.96	No	-
At1g29430	SAUR62	Yes	3.00	No	-
At1g29510	SAUR67	Yes	2.93	No	-
At1g29490	SAUR68	Yes	14.36	No	-
At5g20820	auxin responsive protein SAUR, putative	Yes	3.07	No	-
At4g03190	AFB1, TIR1 subfamily protein	Yes	2.11	No	-
At1g14280	PKS2	Yes	2.39	No	-
At5g04190	PKS4	Yes	5.10	No	-
At3g28200	peroxidase, putative	Yes	2.44	No	-
At1g19180	JAZ1	Yes	2.28	No	-
At2g40000	HSPRO2	Yes	2.65	No	-
At3g44260	CCR4-NOT transcription complex protein	Yes	2.17	No	-
At4g08950	EXO	Yes	5.74	No	-
At3g02170	LNG2	Yes	2.87	No	-
At4g38400	ATEXLA2	Yes	3.40	No	-
At2g20750	ATEXPB1	Yes	2.61	No	-

At4g30290	XTH19	Yes	4.64	No	-
At5g57560	XTH22/TCH4	Yes	3.52	No	-
At1g32170	XTH30	Yes	2.24	No	-
At3g62720	XT1	Yes	2.16	No	-
At3g28180	ATCSLC4	Yes	2.34	No	-
At5g22940	F8H, glycosyltransferase family protein	Yes	2.53	No	-
At3g10960	xanthine/uracil permease family protein	Yes	2.29	No	-
At3g25620	ABC transporter family protein	Yes	2.51	No	-
At4g24570	mitochondrial substrate carrier family protein, putative	Yes	3.13	No	-
At3g30180	CYP85A2/BR6OX2	Yes	2.18	No	-
At4g11280	ACS6	Yes	2.76	No	-
At3g14440	NCED3	Yes	7.47	No	-
At1g15550	GA3OX1	Yes	2.37	No	-
At2g18700	ATTPS11	Yes	2.06	No	-
At2g22830	squalene monooxygenase, putative	Yes	4.06	No	-
At2g47440	DNAJ heat shock N-terminal domain-containing protein	Yes	2.41	No	-
At3g25900	HMT-1	Yes	2.43	No	-
At3g28340	GATL10	Yes	3.54	No	-
At4g10020	short-chain dehydrogenase/reductase protein	Yes	19.91	No	-
At4g03140	short-chain dehydrogenase/reductase protein, putative	Yes	4.58	No	-
At5g50130	short-chain dehydrogenase/reductase protein, putative	Yes	3.74	No	-
At4g22780	ACT domain containing proteins	Yes	5.56	No	-
At4g25280	adenylate kinase family protein	Yes	2.50	No	-
At5g18930	BUD2	Yes	2.57	No	-
At1g02660	lipase class 3 family protein	Yes	2.12	No	-
At1g64660	ATMGL	Yes	6.05	No	-
At1g21980	ATPIP5K1	Yes	2.10	No	-
At1g67880	glycosyl transferase family protein	Yes	2.23	No	-
At2g30040	MAPKKK14	Yes	7.64	No	-
At3g19380	PLANT U-BOX 25	Yes	2.01	No	-
At2g31010	protein kinase family protein	Yes	2.41	No	-
At3g44610	protein kinase family protein	Yes	3.11	No	-
At5g59010	protein kinase family protein	Yes	3.84	No	-
At4g25390	serine/threonine/tyrosine kinase	Yes	2.28	No	-
At5g40540	serine/threonine/tyrosine kinase	Yes	2.45	No	-
At5g40630	ubiquitin family protein	Yes	2.27	No	-

At1g66400	calmodulin-related protein, putative	Yes	2.42	No	-
At4g27280	calcium-binding EF hand family protein	Yes	5.88	No	-
At3g06990	DC1 domain-containing protein, putative	Yes	2.32	No	-
At5g66080	protein phosphatase 2C	Yes	3.35	No	-
At5g01210	transferase family protein	Yes	2.05	No	-
At1g31320	LBD4	Yes	2.99	No	-
At2g42800	ATRLP29	Yes	3.57	No	-
At4g00820	IQ-domain 17	Yes	2.50	No	-
At1g13670	unknown protein, putative	Yes	2.50	No	-
At1g27210	unknown protein, putative	Yes	2.30	No	-
At2g27080	unknown protein, putative	Yes	3.25	No	-
At2g28305	unknown protein, putative	Yes	3.82	No	-
At3g29370	unknown protein, putative	Yes	3.84	No	-
At3g42800	unknown protein, putative	Yes	4.83	No	-
At4g09970	unknown protein, putative	Yes	2.64	No	-
At4g39390	unknown protein, putative	Yes	2.01	No	-
At2g44500	unknown protein, putative	Yes	2.40	No	-
At5g02200	unknown protein, putative	Yes	3.21	No	-
At5g06930	hypothetical protein	Yes	3.25	No	-
At5g44250	unknown protein, putative	Yes	2.03	No	-
At5g51670	unknown protein, putative	Yes	2.36	No	-
At3g55840	Hs1pro-like, unknown protein, putative	Yes	7.27	No	-
At1g23340	unknown protein, putative	Yes	4.89	No	-
At1g35140	unknown protein, putative	Yes	6.73	No	-
At1g50040	unknown protein, putative	Yes	2.53	No	-
At2g36220	unknown protein, putative	Yes	2.29	No	-
At2g35290	unknown protein, putative	Yes	2.05	No	-
At3g19680	unknown protein, putative	Yes	2.01	No	-
At3g23170	unknown protein, putative	Yes	2.24	No	-
At3g54000	unknown protein, putative	Yes	2.01	No	-
At3g54510	unknown protein, putative	Yes	2.32	No	-
At4g35320	unknown protein, putative	Yes	2.07	No	-
At5g43180	unknown protein, putative	Yes	2.99	No	-
At5g56980	unknown protein, putative	Yes	2.19	No	-

Expressed in blades ONLY

AGI code	Description	Expressed in petioles?	Induction in petioles - EOD-FR/D	Expressed in blades?	Induction in blades - EOD-FR/D
At1g68810	bHLH030	No	-	Yes	2.12
At2g43060	bHLH158	No	-	Yes	3.07
At3g25710	bHLH032	No	-	Yes	4.32
At5g28300	MYB-like	No	-	Yes	2.12
At3g04030	MYB-like	No	-	Yes	2.45
At5g43700	IAA4	No	-	Yes	2.04
At5g44210	ERF9	No	-	Yes	3.89

At2g28050	pentatricopeptide (PPR) repeat-containing protein	No	-	Yes	3.44
At2g18010	SAUR10	No	-	Yes	2.96
At3g03830	SAUR28	No	-	Yes	3.64
At1g29450	SAUR64	No	-	Yes	3.15
At5g47800	NPH3 family protein, putative	No	-	Yes	2.13
At5g54490	PBP1	No	-	Yes	2.16
At4g39400	BRI1	No	-	Yes	2.09
At3g49120	PEROXIDASE 34	No	-	Yes	2.26
At2g40610	ATEXP8	No	-	Yes	5.23
At2g38120	AUX1	No	-	Yes	2.70
At1g73590	PIN1	No	-	Yes	2.79
At1g70940	PIN3	No	-	Yes	2.61
At3g51670	SEC14 cytosolic factor family protein	No	-	Yes	2.36
At5g46240	KAT1	No	-	Yes	4.19
At4g13260	YUCCA2	No	-	Yes	2.91
At4g28720	YUCCA8	No	-	Yes	17.44
At1g04180	YUCCA9	No	-	Yes	146.49
At5g55250	IAMT1	No	-	Yes	3.65
At4g37770	ACS8	No	-	Yes	10.58
At1g02400	GA2OX4	No	-	Yes	2.90
At4g16770	oxygenase family protein	No	-	Yes	4.02
At5g02540	SRD family protein	No	-	Yes	12.36
At2g45600	hydrolase family protein	No	-	Yes	3.36
At3g53180	N-terminal protein myristoylation	No	-	Yes	2.12
At4g39800	ATIPS1	No	-	Yes	2.21
At3g58640	protein kinase family protein	No	-	Yes	2.36
At2g01150	RHA2B	No	-	Yes	2.48
At2g41820	leucine-rich repeat transmembrane protein kinase	No	-	Yes	3.53
At2g31980	cysteine proteinase inhibitor, putative	No	-	Yes	2.93
At5g52890	AT hook motif-containing protein	No	-	Yes	2.22
At3g26932	DRB3	No	-	Yes	2.29
At5g02890	transferase family protein	No	-	Yes	2.40
At5g48900	pectate lyase family protein	No	-	Yes	4.91
At1g21820	unknown protein, putative	No	-	Yes	2.52
At4g32290	unknown protein, putative	No	-	Yes	2.08
At5g03230	unknown protein, putative	No	-	Yes	2.18
At5g62280	unknown protein, putative	No	-	Yes	5.86
At1g54200	unknown protein, putative	No	-	Yes	3.02
At4g28240	unknown protein, putative	No	-	Yes	2.65
At4g30410	unknown protein, putative	No	-	Yes	2.13
At5g01790	unknown protein, putative	No	-	Yes	3.06
At5g25460	unknown protein, putative	No	-	Yes	2.36
At5g49170	unknown protein, putative	No	-	Yes	2.29

Expressed in both blades and PETIOLES					
AGI code	Description	Expressed in petioles?	Induction in petioles - EOD-FR/D	Expressed in blades?	Induction in blades - EOD-FR/D
At1g02340	HFR1	Yes	6.57	Yes	53.07
At5g07010	ATST2A	Yes	13.50	Yes	65.87
At1g75450	CKX5	Yes	2.37	Yes	9.74
At5g52900	unknown protein, putative	Yes	2.50	Yes	9.69
At5g07000	sulfotransferase family protein	Yes	3.03	Yes	8.89
At4g16780	ATHB2	Yes	10.51	Yes	30.04
At2g42870	PAR1	Yes	2.37	Yes	5.09
At2g21050	LAX2	Yes	2.06	Yes	3.91
At3g06370	NHX4	Yes	2.04	Yes	3.67
At1g18400	BEE1	Yes	2.57	Yes	4.43
At4g16515	unknown protein, putative	Yes	3.11	Yes	5.29
At5g54510	GH3.6/DFL1	Yes	2.68	Yes	4.35
At3g16800	protein phosphatase 2C family protein	Yes	2.53	Yes	3.76
At5g41400	zinc finger (C3HC4-type RING finger) family protein	Yes	3.36	Yes	4.93
At3g63440	CKX6	Yes	5.07	Yes	7.30
At3g15540	IAA19	Yes	11.66	Yes	16.70
At5g08130	BIM1	Yes	2.14	Yes	3.03
At2g39870	unknown protein, putative	Yes	2.11	Yes	2.98
At2g18790	Phytocrome B	Yes	3.08	Yes	4.34
At5g54470	zinc finger (B-box type) family protein	Yes	15.63	Yes	21.11
At5g47370	HAT2	Yes	4.59	Yes	6.09
At2g23170	GH3.3	Yes	31.77	Yes	40.90
At2g28400	unknown protein, putative	Yes	4.37	Yes	5.55
At5g18060	SAUR23	Yes	8.50	Yes	10.70
At1g09250	bHLH149	Yes	2.12	Yes	2.52
At5g60840	unknown protein, putative	Yes	2.35	Yes	2.67
At5g15580	LNG1	Yes	3.06	Yes	3.43
At1g10560	PLANT U-BOX 18	Yes	2.29	Yes	2.57
At4g32280	IAA29	Yes	18.66	Yes	20.00
At2g46570	LAC6	Yes	3.57	Yes	3.83
At1g80280	hydrolase, alpha/beta fold family protein	Yes	2.22	Yes	2.35
At4g09890	unknown protein, putative	Yes	2.44	Yes	2.43
At5g24580	copper-binding family protein	Yes	2.47	Yes	2.42
At5g66590	allergen V5/Tpx-1-related family protein	Yes	6.85	Yes	6.24
At1g14920	GAI	Yes	2.30	Yes	2.09
At1g29440	SAUR63	Yes	5.43	Yes	4.85
At3g28420	unknown protein, putative	Yes	6.69	Yes	5.77
At3g23030	IAA2	Yes	3.08	Yes	2.63
At1g21060	unknown protein, putative	Yes	3.04	Yes	2.52
At1g69010	BIM2	Yes	2.44	Yes	2.01
At3g55740	PROT2	Yes	4.15	Yes	3.41
At3g03840	SAUR27	Yes	6.47	Yes	5.07
At2g26710	CYP734A1/BAS1	Yes	7.65	Yes	5.62

At1g21050	unknown protein, putative	Yes	6.07	Yes	4.26
At4g25260	invertase/pectin methylesterase inhibitor family protein	Yes	4.56	Yes	3.09
At3g29575	unknown protein, putative	Yes	5.52	Yes	3.58
At3g50340	unknown protein, putative	Yes	5.00	Yes	3.15
At2g22810	ACS4	Yes	7.84	Yes	4.89
At4g14560	IAA1	Yes	4.82	Yes	2.94
At1g29460	SAUR65	Yes	5.45	Yes	3.27
At1g29500	SAUR66	Yes	5.01	Yes	3.00
At5g12050	unknown protein, putative	Yes	9.17	Yes	5.42
At1g15580	IAA5	Yes	19.19	Yes	11.21
At5g02580	unknown protein, putative	Yes	55.98	Yes	29.65
At5g44260	zinc finger (CCCH-type) family protein	Yes	5.47	Yes	2.66
At3g62100	IAA30	Yes	17.33	Yes	7.66
At4g27260	GH3.5	Yes	15.47	Yes	6.18
At3g59900	ARGOS	Yes	8.49	Yes	3.28
At5g02760	protein phosphatase 2C family protein	Yes	23.13	Yes	8.82
